# Nutrition for Children and Adolescents Who Practice Sport: A Narrative Review

**DOI:** 10.3390/nu16162803

**Published:** 2024-08-22

**Authors:** Maria Elena Capra, Brigida Stanyevic, Antonella Giudice, Delia Monopoli, Nicola Mattia Decarolis, Susanna Esposito, Giacomo Biasucci

**Affiliations:** 1 Pediatrics and Neonatology Unit, Guglielmo da Saliceto Hospital, 29121 Piacenza, Italy; m.capra@ausl.pc.it (M.E.C.); g.biasucci@ausl.pc.it (G.B.); 2 Pediatric Clinic, Department of Medicine and Surgery, University of Parma, 43126 Parma, Italy; 3 Department of Medicine and Surgery, University of Parma, 43126 Parma, Italy

**Keywords:** sport activity, agonist, non-agonist, physical activity, childhood, adolescence, nutrition

## Abstract

At a developmental age, adequate physical activity is fundamental to overall health and well-being and preventing obesity. Moreover, establishing active behavior can help children and adolescents meet their growth and neurodevelopmental goals. Nutritional requirements vary according to intensity, frequency, and practiced physical activity or sport; therefore, pediatricians should give children and adolescents and their families adequate counseling, avoiding both nutrient deficiencies and excessive or inadequate supplement intake. The focus should be not only on sports performance but also on the child’s well-being, growth, and neurodevelopment. Our narrative review aims to discuss the nutritional needs of children and adolescents who practice physical activity, non-competitive sports activity, and elite sports activity while also analyzing the role of food supplements and the risk of eating disorders within this category of subjects.

## 1. Introduction

At a developmental age, adequate physical activity is fundamental to ensure overall health and well-being and to prevent overweight and obesity [1]. Establishing active behavior can help children and adolescents meet their growth and neurodevelopmental goals [2,3]. Sports practice is encouraged as well, and many subjects, especially during adolescence, start practicing sports at an *elite* and competitive level. In this context, nutrition plays a pivotal role in supporting children’s and adolescents’ growth and development. Nutritional requirements vary according to the intensity, frequency, and kind of practiced physical activity or sport; therefore, pediatricians should give children and adolescents and their families adequate counseling, avoiding both nutrient deficiencies and excessive or inadequate supplement intake. The focus should be not only on sports performance but first on the child’s well-being, growth, and neurodevelopment.

Our narrative review aims to discuss the nutritional needs of children and adolescents who practice physical activity, non-competitive sports, and *elite* sports activity.

## 2. Methods

The MEDLINE–PubMed database was searched to collect and select publications from 1990 to 2024. The search included randomized placebo-controlled trials; controlled clinical trials; double-blind, randomized controlled studies; and systematic reviews. The following combinations of keywords were used: “nutrition” OR “nutritional habits” AND “children” OR “adolescents” OR “pediatric” AND “physical activity” OR “non-competitive sports activity” OR “*elite* sport” OR “*elite* sports activity” OR “eating disorders” OR “dietary patterns” OR “food supplements” OR “energy drinks” OR “sports drinks”. We also conducted a manual search of the reference lists of the selected studies. The search was limited to English-language journals and full papers only.

## 3. Physical Activity, Sport, and Athletes

When dealing with physical and sports activities, it is important to use correct, validated, and shared terms. The World Health Organization (WHO) defines active people as “individuals and/or groups who integrate physical activity into daily routines”. Life can be considered active if global recommendations on physical activity are met through practices such as walking, cycling, playing, gardening, and other activities that can be considered physical activity [4]. Physical activity is defined as “any bodily movement produced by skeletal muscles that requires energy expenditure”, including those experienced during leisure time, for transport to get to and from places, or as part of daily working activity. Common ways to be physically active involve walking, cycling, recreation, play, and sports, which can be performed at any skill level [5]. On the contrary, physical inactivity is “an insufficient physical activity level to meet present physical activity recommendations”. Sedentary behavior is defined as “any waking behavior characterized by an energy expenditure of 1.5 Metabolic Equivalent of Task or lower while sitting, reclining, or lying. Most desk-based office work, driving a car, and watching television are examples of sedentary behaviors; these can also apply to those unable to stand, such as wheelchair users”. The term exercise, instead, refers to a subcategory of structured, planned, targeted, and repetitive physical activity, in the sense that the improvement or maintenance of one or more components of fitness is the goal. The terms “exercise” and “training” are often used interchangeably and usually refer to physical activity performed during leisure time, with the main aim of preserving or improving health, physical performance, or physical fitness. The term “sport” includes a wider range of activities that are carried out within a set of rules, whether in competition or for leisure. Sports activities consist of physical activity performed by individuals or teams and may be supported by an institutional framework, such as a sports agency. The term “fitness”, on the other hand, describes “the ability to carry out daily tasks with vigor and alertness, without undue fatigue, and with ample energy to enjoy leisure-time pursuits and respond to emergencies. Physical fitness includes several components, including cardiorespiratory, endurance (aerobic power), skeletal muscle endurance, skeletal muscle strength, skeletal muscle power, flexibility, balance, speed of movement, reaction time, and body composition” [4]. Physical activity recommendations for adult subjects and children and adolescents are different, due to the different physical and developmental characteristics of these groups. This study considers the physical activity guidelines and references the latest WHO guidelines [6].

Etymologically, the term athlete derives from the Greek root “Athlos”, which means “competition” or “achievement”. It has been reported that the first documented use of this term is found in *The Odyssey*, when the Faeci accused Ulysses of lacking virtue, being greedy and therefore not being an athlete, as he had refused to compete in sports contest [7]. Nowadays, echoing this concept, different organizations, including the European Society of Cardiology (ESC) [8] and the American Heart Association (AHA) [9], agree in emphasizing “organized competition” and “award for excellence and success” as key components in the definition of an athlete. In fact, while the ESC defines an athlete as “an individual of young or adult age, either amateur or professional, who is engaged in regular physical training and participates in official sports competitions” [8], the AHA defines him/her as “one who participates in organized team or individual sports that require regular competition against others as a fundamental component, and who places a high value on excellence and achievement, requiring some form of systematic (usually intense) training” [9]. In 2016, Araújo et Scharhag proposed that, to be considered an athlete, four criteria must be fulfilled simultaneously, to standardize and modernize the use of the term athlete for medical and health sciences research [10].

These criteria were later endorsed and revised by Mac Mahon C. and Parrington L., who defined athletes as “people who engage in physical activity with the primary goal of improving performance to bolster athletic excellence and/or achievement” [11]. Therefore, individuals who do not fulfill these criteria, but who regularly practice sport or physical activity, or even compete and train alone or with friends and/or teammates, should be called exercisers and not athletes. This term should substitute obsolete expressions such as recreational athletes or amateur [12]. In 2019, McKinney et al. argued that the training intent should be a major criterion for distinguishing an athlete from an exerciser. If exercisers prioritize promoting health (including body aesthetics) over performance, the opposite can be said for athletes. In addition, the authors suggest that for the better stratification of athletes, their “training volume” (hours per week) and “competition level” should also be considered—see Table 1 [13].

## 4. Nutritional Advice for Children and Adolescents Who Practice Physical Activity

Regular physical activity offers numerous advantages to children and adolescents: enhanced social engagement, heightened physical well-being, and the fostering of self-concept and self-worth. Adolescence represents a crucial phase marked by substantial physical transformations, characterized by shifts in body composition, metabolic dynamics, hormonal variations, organ system maturation, and the establishment of nutrient reserves, all of which can significantly influence future health outcomes [14]. Engagement in sports prominently contributes to bolstering psychological well-being and cultivating a positive self-perception in most adolescents [15]. Optimal nutrition can enhance performance, prevent injuries, expedite post-exercise recovery, promote healthy lifestyle practices, sustain overall wellness and appropriate weight management [16].

In the developmental age, it is fundamental to establish adequate nutritional habits, both from a qualitative and a quantitative point of view, to support growth and development, and to grant adequate energy needed for physical activity, training, and competition [17]. Although there are general estimates of energy expenditure for adolescent athletes (such as approximately 3640 ± 830 kcal/day for males and 3100 ± 720 kcal/day for females), the actual energy needs of individual adolescent athletes can vary significantly [18]. Variations in training intensity, competition demands, involvement in multiple sports, part-time work, and sedentary behaviors can affect the energy needs of adolescent athletes. Determining individual energy requirements is additionally complicated by metabolic and hormonal differences among individuals, as well as challenges in accurately estimating both energy intake and expenditure [19,20]. The energy requirements for growth, which are a crucial aspect of the energy needs of adolescent athletes, encompass two components: the energy used to create new tissues, and the energy stored in growing tissues. The energy expended in tissue synthesis can be directly assessed using the doubly labeled water (DLW) method, which is an isotope-based technique for the assessment of energy expenditure, based on the difference between the turnover rates of the hydrogen and oxygen of body water as a function of carbon dioxide (CO_2_) production [21], or, more frequently, estimated indirectly through measurements of the resting metabolic rate (RMR) [22]. Guidelines regarding various intensity levels of physical activity (PA) should be complemented by proper nutrition, and, conversely, nutrition should be aligned with PA recommendations. Physical activity typically contributes to approximately 25 to 30% of the total daily energy expenditure in a non-athletic individual and varies based on the intensity of PA [23]. The maximum oxygen uptake (VO_2_ max) is an objective measure of cardiorespiratory fitness. It must be assessed individually for each activity, although its widespread use is limited due to associated costs. During aerobic exercises, VO_2_ increases until it reaches a stable value, which is reached earlier in children aged 10 to 11 years compared to adults, and it varies based on activity levels [23]. Energy requirements vary according to age; developmental age can be divided into three distinct age brackets: toddlers (under 3 years old), young school-age children (aged 3–12), and teenagers (aged 13–19). Within each of these categories, the energy expended for growth and the level of physical activity (referred to as physical activity level [PAL]) are considered, with PAL values set at 1.40, 1.58, and 1.75, respectively. These figures, combined with basal metabolic rates (adjusted for age group, gender, height, and weight), serve as the basis for estimating the average requirements within each age group. However, individuals within each age bracket may deviate from the standard PAL associated with their group. In such instances, energy requirements will increase accordingly. For instance, children engaging in 30 min of moderate physical activity on five or more days per week or participating in 60 min of vigorous sports five times weekly, or those undertaking intense aerobic exercises linked to competitive sports, will experience a PAL adjustment of 0.15, 0.3, or 0.6 units, respectively [24]. Moreover, research has highlighted an inverse correlation between PAL and body mass percentage in both young children and adolescents, especially among males. Consequently, children engaging in prolonged, intense physical activity, such as 2 h of accumulated performance, have exhibited a more significant decrease in body fat levels compared to those with lower levels of activity [25].

### 4.1. Carbohydrates

Carbohydrates (CHOs) are the main energy source for the muscles and brain. Insufficient CHO intake can lead to premature fatigue, prompting the body to use muscle tissue for energy. Additionally, young subjects typically store less glycogen compared to adults, potentially resulting in a quicker onset of fatigue during physical activity [26]. Adequate CHO sources should be carefully chosen during training periods. It is important to dispel the misconception that CHOs are solely derived from grains. Fruits, milk, yogurt, and starchy vegetables, such as legumes, potatoes, and winter squash, also serve as valuable carbohydrate sources. When practicing physical activities, the duration and intensity of exercise sessions determine the utilization patterns of CHOs and the subsequent refueling needs [27]. The recommended CHO intake (according to dietary reference intakes [DRIs]) for a child engaged in normal physical activity typically falls within the range of 50 to 55%, equating to approximately 6 to 10 g per kilogram of body weight [17]. At least fifty percent of the calorie intake for children involved in high-performance sports should be derived from CHOs, distributed evenly throughout the day. In the case of exceptionally intense exercise, the total CHO intake should escalate to around 70%. Adequate CHO intake is particularly crucial on competition days, where the focus lies on replenishing muscle glycogen stores in the hours leading up to the event, and sustaining performance throughout its entirety, especially if it extends beyond one hour. Following the conclusion of the activity, the child or adolescent needs to consume a meal that prevents post-exercise muscle breakdown. The ideal meal should provide 4 g of CHO per kilogram of body weight in the 3 to 4 h preceding the sports activity, followed by 0.5 to 1 g per kilogram in the hour preceding it. During the activity, a consumption rate of 0.7 g per kilogram every hour, distributed in 15 to 20 min intervals, is recommended. Post-activity, 1 to 1.5 g of CHO intake per kilogram is advisable [18]. Dietary approaches utilizing refined CHOs (e.g., sports drinks, gels, and bars) to modify metabolism during endurance sports, like CHO loading or employing multiple transportable forms of CHO during exercise, are expected to boost performance in events exceeding 90 min. However, it is fundamental to apply these recommendations only when suitable. Additionally, it is essential to consider that the excessive utilization of these tactics may heighten the risk of both tooth decay and obesity.

### 4.2. Fats

Adequate dietary fat intake is essential to provide energy to support growth and maturation and to fulfill the requirements of fat-soluble vitamins and essential fatty acids [28]. Furthermore, maximal fat oxidation rates, relative to lean mass, are slightly elevated in athletes younger than 18 years old [29]. Recent guidelines suggest a dietary fat intake comprising 20–35% of total energy while ensuring that saturated and trans fatty acids together do not exceed 10% of total energy intake [30,31]. Fats are the optimal fuel source for low-intensity and long-duration activities. Physical exercise can result in an elevation of fat utilization and mobilization. Lipid oxidation in the form of free fatty acids is slow. About 20 to 30 min after exercise begins, the body starts using free fatty acids as a steady source of energy. There is no solid proof that taking extra fat supplements improves performance in sports. Normally, the body’s stored fat is enough to meet energy needs [30,31]. Generally, children aged between 1 and 18 years should have a fat intake ranging from 25 to 35% of their daily calories. Saturated fats should account for less than 10% of total fat intake, while polyunsaturated fats may contribute up to 10%, including 1 to 2% from linoleic acids. Furthermore, 10 to 15% of fat intake should consist of monounsaturated fats, and daily cholesterol consumption should be limited to 300 mg [32]. However, sports nutrition recommendations advocate for athletes to adjust their dietary intake in response to changing energy demands, to enhance daily performance and maximize adaptations to training [33]. This principle is commonly referred to as “fuel” for the work required [34].

### 4.3. Proteins

Children and adolescents need adequate protein intake to promote overall growth and development, as well as to improve their response to exercise training [32]. Protein intake is crucial to support growth and to meet the demands of intense exercise or high-stress situations. Proteins play a key role in muscle synthesis and repair, and they should contribute to 10 to 15% of the total daily caloric intake [25]. The main protein sources are eggs, beef, chicken, fish, and cow’s milk. Protein requirements are heavily influenced by total energy intake. In case of insufficient energy intake, endogenous protein and liver glycogen are mobilized to regulate blood glucose levels, potentially reducing the availability of protein for their essential functions. Consuming approximately 0.11 g of protein per kilogram per hour during post-exercise recovery, or roughly equivalent to 1.5 g per kilogram per day (such as approximately 0.3 g of protein per kilogram at five mealtimes), should effectively replenish any exercise-induced amino acid oxidative losses, enhance overall net protein balance, and support the normal growth and development of adolescent athletes [35,36]. Protein supplements are not necessary for children and adolescents who practice physical activity. On the contrary, excessive protein intake does not enhance muscle mass but may lead to an increase in the fat compartment. Additionally, it can elevate the risk of health issues associated with a diet deficient in other essential nutrients [32]. Finally, proteins should be consumed within 30 min after exercise, and again within the following 2 h to help restore glycogen stores and allow muscles to recover [37]. After exercise, the co-ingestion of CHOs and proteins is associated with higher glycogen synthesis rates than CHO intake alone [38].

### 4.4. Hydration

Adequate fluid intake is crucial for subjects who practice physical activity, to prevent dehydration and maintain normal cardiovascular and thermoregulatory functions. Children are more susceptible to heat stress compared to adults, as they have a higher surface-area-to-body-mass ratio, resulting in increased heat absorption from the environment. Additionally, children produce more metabolic heat per unit of mass during physical activities such as walking or running. Moreover, children have a lower sweating capacity, which limits their ability to dissipate body heat through evaporation. Hydration should be maintained during physical activity, not only to mitigate the risk of heat-related illnesses but also to optimize performance. The fluid intake recommendations for children and adolescents who practice physical activity [28,39] closely resemble those advised for adults [40]. Exercise should be started in optimal hydration conditions, crafting personalized hydration strategies (adjusted periodically during puberty to adapt to changes in sweat rate), restricting body mass reduction during activity to ≤2% from pre-exercise levels, and discouraging weight gain. A fluid intake of 13 mL/kg per hour of exercise should be satisfactory to prevent significant fluid deficits in developing athletes [39]. In the case of activities lasting less than 60 min, water is enough. However, in case of exercises exceeding this duration, particularly in extremely hot and humid conditions, consuming CHOs during the activity may offer additional benefits [41]. Consuming sports drinks during exercise offers a convenient way to replenish CHOs, fluids, and electrolytes. Yet, drinking sports drinks when not active can lead to excessive caloric and CHO intake, which might lead to dental problems, weight gain, and obesity [42]. Sports drinks and energy drinks are not the same thing. A sports drink is usually composed of CHOs, minerals like sodium, potassium, calcium, and magnesium, and sometimes vitamins or other nutrients. On the other hand, an energy drink usually contains stimulants like caffeine, green tea extract, and guarana, along with CHOs, amino acids, vitamins, and minerals [42]. So, non-athletic individuals should avoid the regular consumption of sports drinks with CHOs, as they can contribute to excessive calorie intake, increasing the risk of weight gain and tooth decay [14,15,16,42].

## 5. Nutrition for Young *Elite* Athletes

In the past few decades, the number of children and adolescents involved in competitive sports has increasingly grown [43]. The terms “competitive” and “*elite*” refer, generally, to an athlete who achieves the highest level of competition and trains more than 10 h a week [13].

### 5.1. Energy Requirements

Nutrition plays a key role in athletic performance, especially during adolescence. To address the unique nutritional needs of adolescent athletes, Sports Dietitians Australia (SDA) issued a position statement titled “Sports Nutrition for the Adolescent Athlete.” It is difficult to define the appropriate daily energy intake of young *elite* athletes since energy expenditure is mainly influenced by the training and competition load [43]. In a report published by the Food Agriculture Organization (FAO), the World Health Organization (WHO), and the United Nations University (UNU), energy requirements for boys and girls participating in heavy physical activity are defined [13]. The daily energy intake increases with age and is different between boys and girls from pubertal age, as shown in Table 2.

### 5.2. Macronutrients

#### 5.2.1. Carbohydrates

CHO is used by athletes as a primary substrate to fuel daily exercise, and young athletes are encouraged to assume at least 50% of their diet in the form of CHOs [26]. According to Burke et al., during an endurance exercise program (1–3 h/day), athletes need 6–10 gr/kg/day of CHOs, whereas during an extreme exercise program (4–5 h/day), the CHO intake required is 8–12 gr/kg/day. During the sports session, if the duration is between 0 and 75 min, no extra CHO or very small amounts of CHO are required, whereas if the duration is between 75 min and 2.5 h, a CHO amount of 30–60 gr/h can be helpful. Furthermore, after exercise, athletes should assume 1–1.2 gr/kg for each hour of training to achieve faster recovery [44].

#### 5.2.2. Proteins

Protein requirements in children are higher than in adults to support growth and physical development, since they are essential for the cellular synthesis of different tissues. Athletes have a major protein requirement due to the elevated demand resulting from exercise performance [45]. There is little data on protein demand in *elite* young athletes, and most available studies refer to the adult population. The SDA asserted that a competitive adolescent athlete should follow the *elite* adult athletes’ guidelines regarding protein requirements (around 1.3–1.8 gr/kg/day), since in the majority of adolescent athletes, the daily protein intake (1.2–1.6 gr/kg/day) is consistent with the adult recommendations and there is therefore no need for any protein supplementation to reach a positive nitrogen balance for adolescents participating in heavy exercise [46].

#### 5.2.3. Fats

Fat consumption for competitive young athletes has not been fully investigated. Adolescent fat consumption should not be higher than 30% of daily energy intake, and adolescent competitive athletes are recommended to follow these same public health guidelines; ideally, they should prefer unsaturated fats, which derive from vegetables and fish sources, and limit saturated fats by avoiding processed and fried products [46].

#### 5.2.4. Fluid Intake

Fluid intake is a crucial issue regarding *elite* young athletes’ nutrition. During sports performance, heat loss occurs by the evaporation of sweat, which can result in large fluid and electrolyte losses. An appropriate fluid balance is mandatory to avoid dehydration during exercise. Additionally, we know that children have a higher ratio of body surface area to body mass than adults, which leads to a higher amount of heat dissipation through sweat, and, consequently, there is a higher risk of dehydration. The fluid intake for competitive athletes must be satisfactory, to reduce fluid losses to less than 3% of body mass; the correct amount of liquid to introduce can be obtained by measuring body weights before and after training to predict the sweat rates and thus the fluid losses [47]. A descriptive study showed that most *elite* young athletes in several sports (basketball, gymnastics, swimming, running, canoeing) are often dehydrated, as evaluated through urine examination and Urinary Specific Gravity (USG) analysis. Over 89% of the fifty-nine young male *elite* athletes analyzed were hypo-hydrated before starting practice (USG ≥ 1.020 mg/dL) and during the training day, despite fluid availability. This finding demonstrates that *elite* young athletes mostly adopted inadequate rehydration habits [48,49], which can lead to remarkable changes in athletic performance.

#### 5.2.5. Micronutrients

When practicing sports at an *elite* level, an adequate intake of micronutrients, such as calcium, vitamin D, and iron, is recommended as well [50]. The calcium requirement is higher in young athletes due to bone development. The American Academy of Pediatrics (AAP) suggested a calcium daily intake of 1300 mg for children and adolescents aged from 9 to 18 years old and a vitamin D daily intake of at least 600 UI for children and adolescents aged from 1 to 18 years old, with supplementation, particularly among individuals who are not likely to be exposed to sufficient sunlight [51]. Moreover, in the athletic population, an adequate calcium and vitamin D balance reduces muscle cramps, inflammation, and the risk of stress fractures [25]. Iron is a fundamental micronutrient related to optimal sports performance. Competitive adolescent athletes, and among these, especially female athletes, have a higher probability of having an iron deficiency because of growth, menstruation, or vegetarian and vegan diets. Hence, in case of iron deficiency or anemia, oral iron supplementation is recommended [52].

## 6. Food Supplements for Children and Adolescents Who Practice Sport

Sports nutrition aims to improve physical activity, athletic performance, and recovery by combining knowledge of exercise’s physiological basis and scientific principles of nutrition [53]. Making nutritional choices can be challenging for athletes because they need to ensure adequate muscle growth and development, as well as improve their sports performance. Therefore, athletes often tend to use nutritional supplements to meet their nutritional needs [26]. However, it is important to note that supplements should not be used as a substitute for a healthy diet and athletes should consult with a registered dietitian or other qualified healthcare professional to determine if they need to use supplements and which ones are appropriate for them [54]. A dietary supplement is a nutrient, a non-food compound, a food, or part of it taken alongside a regular diet to achieve a specific health or physical performance benefit. The different forms of dietary supplements are summarized in Table 3, adapted from [55].

There is a growing trend of sports supplement use among young athletes. A study by Jovanov et al. revealed that a significant portion of athletes use sports supplements (82.2%), with a majority (60.6%) being male [53]. With young athletes increasingly participating in major sports events, a lack of research on their supplement habits is a critical gap. This knowledge is crucial for developing educational programs to prevent unnecessary and indiscriminate supplement use [56].

### 6.1. Vitamin D

Lean mass and muscle strength are positively associated with bone mineralization during the growth period. Adolescence represents a crucial time for bone growth (peak bone mass can reach up to 90% by the age of 18) [51,57]. Studies conducted on the pediatric population have not reached an agreement yet [58]. Thams L. et al. evaluated the effects of vitamin D and high-protein dairy product supplementation in children aged 6–8 years during the extended winter period. The intervention group received 25 μg of vitamin D and 300 g/day (six days a week) of skyr-type yogurt (a low-fat yogurt, like Greek yogurt, with a protein/casein ratio like normal protein yogurt) for 24 weeks. Although the study showed an increase in leg strength in girls who received vitamin D supplementation, no significant effects on exercise and muscle strength were shown in healthy children [59]. Skalska et al. investigated the effect of vitamin D supplementation in young soccer players during an eight-week high-intensity training period, analyzing the improvement in terms of technical, physical, and tactical skills during small-sided games. Their study led to non-statistically significant results [60]. On the other hand, large cross-sectional studies have revealed positive correlations between blood levels of a vitamin D marker and handgrip strength in children [61,62]. Vitamin D supplementation is always recommended in the event of deficiency. Serum vitamin D levels are involved in regulating bone mineral density, and adolescents who play sports indoors or during winter may be more exposed to bone stress injuries [63]. Mesquita et al. compared vitamin D levels, bone mineral density, and bone geometry in 32 adolescent athletes (track and field and artistic gymnastics) and 43 young non-athletes. Athletes with higher vitamin D levels (≥27 ng/mL) had higher bone mineral density levels if compared to controls [64]. Young athletes, especially those with limited sun exposure, are vulnerable to vitamin D deficiency or insufficiency, which can negatively impact their bone development during adolescence; therefore, it is essential to supplement vitamin D if necessary.

### 6.2. Sport Drinks

Evidence regarding the use of sports drinks among adolescent athletes is still limited. Sports drinks fall among sugar-sweetened beverages and can be an aid during prolonged physical exertion to help individuals stay hydrated and restore blood glucose levels. On the other hand, excessive consumption of such drinks can lead to weight gain, increased obesity, and the risk of dental caries [65]. According to some studies, sports drinks are widely consumed by athletes, but Cordrey et al. have pointed out that their consumption is also very common among adolescents who lead sedentary lives. In agreement with the American Academy of Pediatrics, adolescents engaged in regular physical activity do not need to reintroduce electrolytes through sports drinks, and water must be considered the primary source of hydration [66].

### 6.3. Creatine

Creatine is a substance naturally present in our bodies, which can also be obtained through diet. Creatine’s main food sources are red meat and fish. Creatine is essential to provide energy to our muscles and it represents an important ergogenic aid among athletes. Although several studies and trials have been conducted in adult subjects, in the past two decades, the use of this supplement has also increased among adolescents [67], who mainly use creatine to improve their athletic performance, strength, and muscle mass [68]. However, there is a lack of randomized controlled trials (RCTs) on the safety of creatine use in adolescents. This has led some professionals to hesitate in recommending it for this age group. Recent studies have focused on two specific groups of young athletes: soccer players and swimmers [67]. Several studies demonstrate that creatine can be a valuable ally in improving soccer players’ performances. Ostojic et al. analyzed the effect of creatine supplementation on specific soccer skills. A 7-day supplementation cycle led to improvements in dribbling time, power-sprint performance, and vertical jump, with significant differences between the creatine and placebo group [69]. On the other hand, Yañez-Silva showed a significant difference only for the increase in total work in soccer players who received creatine supplementation. The increase in peak power and average power did not differ significantly, and there were no differences in fatigue between the two groups [70]. The relationship between creatine and swimming is still uncertain. Further studies are needed to better understand its effects on several variables, such as distance traveled, the type of swim, and the individual characteristics of athletes. Juhàsz et al. showed that creatine led to increased power, improved time in the 100 m, and reduced post-exercise lactate. In addition, an increase in muscle mass was observed [71]. A single study conducted by Wang et al. evaluated the effect of creatine on young canoeists, showing that creatine can increase maximal upper-body muscle strength without affecting explosive power or post-activation potentiation time [72].

### 6.4. Caffeine

Caffeine is a methylxanthine alkaloid that exerts psychotropic effects by binding to adenosine receptors in the central nervous system. Its positive effects, which are not consistent across all studies, include improvements in muscle endurance, aerobic and anaerobic performance, speed, muscle strength, sprints, throws, and jumps. The recommended daily intake is 3–6 mg/kg, regardless of age or size. Caffeine is one of the main ingredients in energy drinks and shots, and teens (12–18 years old) should be careful about consuming them. It is best to talk to a parent or guardian first, especially if they plan to consume more than 400 milligrams daily. There is not enough information yet to know if these drinks are safe for teenagers. Young children (ages 2–12) should not have energy drinks or shots at all [73].

### 6.5. Polyunsaturated Fatty Acids

Polyunsaturated fatty acids (PUFAs) can be divided into omega-3 and omega-6, according to their biochemical structure. Linoleic acid (LA) and alpha-linolenic acid (ALA) are essential fatty acids, so they must be introduced with diet and cannot be synthesized de novo by humans. The main ALA sources are vegetable oils, nuts and seeds like walnuts and flaxseeds, wild fish, and microalgae (the main source of omega 3). The most important fatty acids are eicosapentaenoic acid (EPA), docosapentaenoic acid (DPA) and docosahexaenoic acid (DHA) deriving from ALA, and arachidonic acid (AA) deriving from LA [74].

Omega-3 fatty acids, known for their positive effects on overall health, can also be used to improve sports performance and recovery, and they reduce the risk of injury. The lack of rigorous scientific studies on the use of omega-3 supplements among pediatric athletes prevents definitive conclusions about their efficacy and safety in this specific population. Despite their potential beneficial properties for cardiovascular health and cognitive function, scientific evidence to support their use in young athletes is currently lacking. Further research is needed to determine whether omega-3 supplementation can offer concrete benefits to pediatric athletes in terms of sports performance, injury prevention, or overall health improvement.

Research on the prevalence of sports supplement use in young athletes yields inconsistent findings across different studies. It appears that sports supplement use increases markedly with age and is more prevalent among male athletes. A recent study by Svantorp-Tveiten et al. examined supplement use among high school students (16–18 years old). They found that many young boys regularly took supplements like protein powder (29.4%), creatine (16.7%), and other dietary supplements (7.9%), whereas their use among females is less common, ranging between 3 and 9.5% [75]. Caraballo et al. showed that dinghy sailors mostly supplement isotonic bars and drinks to restore hydration and micronutrients during competitions. Improved performance is the main reason why both males and females use sports supplementation during competitions. In general, males aim for physical and performance improvement, while females aim for health and the prevention of nutritional deficits [76]. Newbury et al. analyzed supplements used in a high-performance swimming club with three talent stages, and 43 out of 44 swimmers reported using at least one dietary supplement. National swimmers (older and more experienced swimmers with higher performance) mainly used individual health supplements (vitamin D3, omega-3 fatty acids, multivitamins, zinc), followed by sports supplements (mainly protein powder) and ergogenic aids (caffeine, creatine, beta-alanine, and sodium bicarbonate). The age group and development swimmers consumed mostly sports supplements, with the highest use of sports drinks among development swimmers. The use of ergogenic aids was significantly lower (age group) or almost absent (development swimmers) in these two groups. Increasing supplement intake was directly proportional to age and type of performance. Performance and recovery were the main reasons for taking supplement. Some counted healthy and convenient nutritional sources. Female swimmers consume supplements mainly for performance enhancement, while male swimmers consume them for muscle growth [77].

The lack of good sources of information when deciding to use supplementation is another problem that needs to be addressed. According to Jagim et al., adolescents indicate teachers and parents as primary sources of information concerning nutritional supplements [73]. Newbury et al. also showed that the main source of information is parents (42%), followed by nutritionists (33%), with a small percentage relying on advice from instructors (6%) [77]. Other research identifies coaches as the primary source of information regarding sports supplements. However, it highlights that teenagers nearing adulthood (aged 17–18) tend to rely more heavily on the internet for guidance on this topic [53]. Further research is required to fully understand the effects of sports supplements on children. This research should involve larger sample sizes and longer observation periods. Pediatricians need to stay up to date on the latest information to provide accurate guidance on whether to recommend sports supplements, which are now widely used among adults.

## 7. Sport and Special Diets

### 7.1. Eating Behaviors in Young Athletes

The effective eating behavior of competitive young athletes has been investigated by several authors. Smith et al. analyzed a sample of 87 *elite* male adolescent rugby players, aged from 14 to 19 years, showing that their dietary profile met the current guidelines on energy, macronutrient, and fluid intake [26]. Burrow et al. analyzed a cohort of Australian adolescent competitive rugby players, with a median age of 16 years old, and they found that they had a “good” dietary practice overall, despite a lower CHO intake (48% of total daily energy; 3.6 g/kg/day) and an elevated intake of processed foods (38% of total daily energy) rich in sugar and salt, such as sugary drinks, packaged snacks, confectionary and takeaway foods [78]. Braun et al. evaluated the nutritional status of 56 young female *elite* football players in Germany, underlining a suboptimal nutrition status, especially for CHO and protein intake, associated with iron and vitamin D deficiency [79]. A systematic review and meta-analysis stated that athletes involved in heavy physical activity tend to have healthier dietary habits compared to non-athletes or young adolescents with lower athletic participation. However, competitive athletes tend to consume fewer essential minerals and have a suboptimal CHO and calorie intake [80]. A study on different energy intakes depending on the nature of endurance athletes’ training analyzed food consumption ad libitum to evaluate the daily energy intake of 14 adolescent males, with an age range from 15 to 16 years old, from an *elite* rugby center. The authors highlighted an increasing energy intake after an exercise session (morning session of cycling) compared to a rugby session (traditional rugby training session) (*p* < 0.01) and a control session (inactivity throughout the day) (*p* < 0.001). Therefore, young competitive athletes experience different nutritional adaptations based on their training intensity [81].

### 7.2. Relative Energy Deficiency in Sport

Recently, a 3-year longitudinal study aimed at investigating relative energy deficiency in sport (REDs) among adolescent male *elite* athletes and its impact on sports performances reported that adequate energy availability (EA) among athletes may be correlated to an appropriate nutritional intake and recovery during the observational period [82].

### 7.3. Eating Disorders in Young Athletes

At the *elite* sport level, eating disorders (EDs) are more common than in the general population. The prevalence of EDs is higher among female athletes compared to male athletes. Male athletes usually want to gain weight through muscle mass, whereas female athletes want to reduce weight and gain a slim appearance. Female *elite* athletes have a higher risk of developing the “female triad”—a term that refers to the simultaneous presence of EDs, amenorrhea, and osteoporosis or osteopenia [83]. The prevalence of the female athlete triad has been investigated in *elite* swimmers aged from 11 to 19 years old in Rio de Janeiro, Brazil. This cross-sectional analysis showed that 35 of 78 athletes (44.9%) appeared positive on at least one of the three questionnaires chosen to detect EDs [84]. *Elite* adolescent female figure skaters are at risk of EDs as well, as reported in another cross-sectional study. The authors showed that, despite an appropriate weight-for-height figure, 24% of a sample of 36 competitive female figure skaters had a clinically significant ED [85]. This trend has been also confirmed by a descriptive and cross-sectional study carried out on 69 *elite* handball players, aged from 15 to 35 years old, which demonstrated that 11% of females could develop an ED, in contrast to 3% of males [86]. It seems clear that young endurance athletes have a higher probability of developing EDs due to mental health problems such as increasing social pressure and body dissatisfaction. Nutritional knowledge plays a key role in the establishment of nutritional habits; correct nutritional education is closely related to healthy food choices. Families, dietitians, physiotherapists, coaches, and the internet represent the main sources of nutritional information for young *elite* athletes. Several studies have been conducted to investigate the nutritional knowledge level among young endurance athletes. In 2015, Alaunyte et al. demonstrated a strong correlation between nutritional knowledge and diet quality in a cohort of *elite* rugby league players, with a median age of 25 years [87]. Vázquez-Espino et al. analyzed the eating behaviors of *elite* football, basketball, and hockey players aged from 13 to 25. They reported a weak but significant correlation between nutritional knowledge of fruits and vegetables and their dietary daily intake (*p* = 0.270) [88]. Nutritional knowledge seems to be not influenced by age, level of education, athletic caliber, or team/individual sport participation, as shown in a study conducted by Spronk et al. on *elite* athletes aged over 16 [89]. In 2016, a questionnaire on nutrition knowledge for young and adult athletes (NUKYA) was developed to establish a compact reliable tool of investigation that allows us to learn about nutritional information known by large groups of athletes [90]. The role of nutritional education has been discussed in a study conducted among young, male Chinese *elite* soccer players. They found that a four-week program on good nutrition helped male Chinese soccer players improve their food and nutrition knowledge [91].

## 8. Conclusions

Physical activity and sports activity are fundamental for children’s and adolescents’ overall well-being and health. Nutrition plays a pivotal role in meeting adequate energy, macronutrients, and micronutrient requirements during physical and sports activity at developmental ages. Children and adolescents who practice physical activity must follow nutritional guidelines appropriate for their age, and no additional supplements are usually needed to support their activity, provided that adequate hydration is granted. The nutrition of *elite* athletes, on the contrary, requires multidisciplinary support by families, coaches, dietitians, nutritionists, and physiotherapists to ameliorate physical and physiological performance while respecting age-specific nutritional and energy requirements. Close nutritional counseling should be mandatory to avoid health problems and to reach ideal sports performance. Supplements and sports drinks can be considered for *elite* athletes but only in selected cases and under nutritional and medical control, to avoid dangerous self-prescriptions.

## Figures and Tables

**Table 1:** Athletes’ stratification according to McKinney et al. [13].

	Training Volume	Competition Level
*Elite* athletes	>10 h/week	Their athletic performances have achieved the highest level of competition
Competitive athletes	>6 h/week	Emphasis on improving performance
Recreational athletes	>4 h/week	Unregulated competitions
Exercisers	>2.5 h/week	Maintain health and fitness status

**Table 2:** Daily energy requirement in children and adolescents with heavy physical activity (adapted from [13]).

AGE (Years)	DAILY ENERGY REQUIREMENT (kcal/day)	
	BOYS	GIRLS
6–8	1800–1950	1650–1775
8–10	2100–2275	1950–2125
10–12	2475–2700	2300–2475
12–14	2925–3175	2625–2725
14–16	3450–3650	2855–2875
16–18	3825–3925	2875

**Table 3:** Type of food supplements used during sports activity.

Type of Food Supplement	Characteristics
Formulate and Sports Foods	These products offer a quick and easy way to obtain essential nutrients without requiring preparation (e.g., meal replacement shakes, protein bars) and are designed to support athletes’ energy needs and recovery before, during, and after exercise (e.g., sports drinks, gels, energy bars).
Functional Foods	These foods have additional nutrients or ingredients beyond what is naturally present to offer specific health benefits (e.g., fortified cereals, protein-enriched yogurt).
Single Nutrients and Herbal Products	Individual vitamins, minerals, or other components extracted from food sources (e.g., vitamin D supplements, iron) and concentrated forms of plant-based ingredients believed to offer health benefits.
Multi-Ingredient Products	They combine various ingredients, like vitamins, minerals, herbs, or other functional components, to target specific health goals (e.g., multivitamins).

## Data Availability

Not applicable.

## Abbreviations

AA	Arachidonic acid
AAP	American Academy of Pediatrics
AHA	American Heart Association
ALA	Alpha-linolenic acid
CHO	Carbohydrate
LA	Linolenic acid
DPA	Docosapentaenoic acid
DHA	Docosaexaehnoic acid
DLW	Doubly labeled water
ED	Eating disorder
EPA	Eicosapentaenoic acid
ESC	European Society of Cardiology
NUKYA	Nutrition knowledge for young and adult athletes
PA	Physical activity
PALI	Physical activity level
PUFA	Polyunsaturated fatty acids
RCTs	Randomized controlled trials
RE	Restrictive eating
REDs	Relative Energy Deficiency in Sport
RMR	Resting metabolic rate
USG	Urinary specific gravity
WHO	World Health Organization
